# Estimating the Bias of Model Compounds for the Determination of Species-Specific Protonation Constants

**DOI:** 10.1021/acsomega.3c06994

**Published:** 2023-12-28

**Authors:** Tamás Pálla, Zsolt Kolompár, Károly Mazák, Arash Mirzahosseini, Béla Noszál

**Affiliations:** Department of Pharmaceutical Chemistry, Semmelweis University, Budapest 1085, Hungary

## Abstract

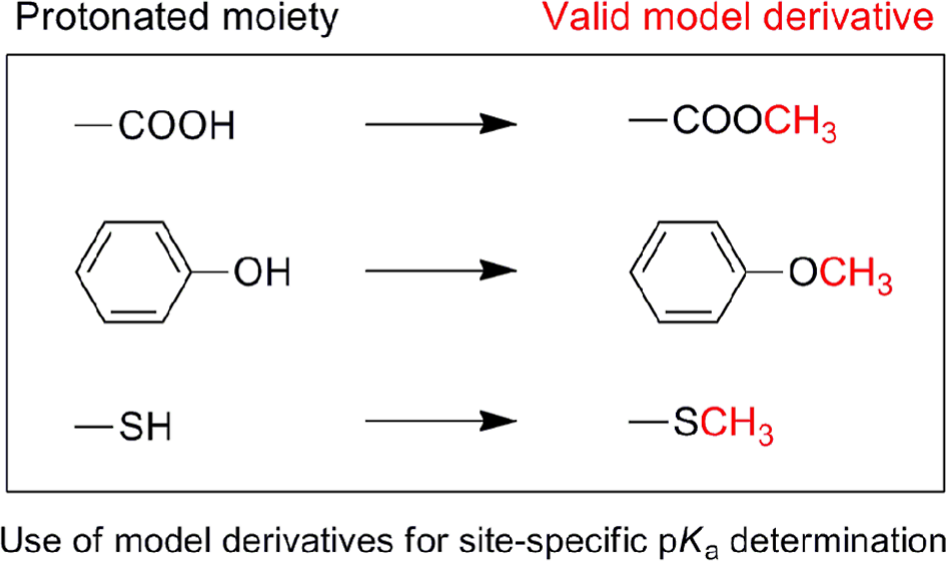

The previously unknown extent of the goodness of using model compounds for the microspeciation of polyprotic systems was studied. Mirror-symmetric dibasic compounds and their monosubstituted derivatives were investigated to quantify how the derivatives are appropriate models of the minor microspecies to be mimicked in various microspeciation systems. The results were analyzed using statistical methods. It was found that the respective O-methyl and S-methyl derivatives of phenols and thiols as well as the methyl esters of carboxylic acids are sufficiently good derivatives for microspeciation. It was also found that the methyl esters are superior to the carboxylic amides for modeling the –COOH moiety.

## 1 Introduction

**Figure 1:**
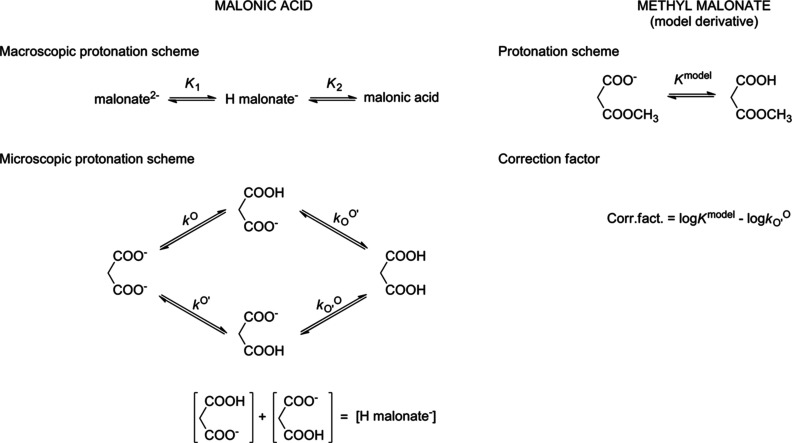
Protonation macro- and microequilibrium schemes of malonic acid (left) and the protonation scheme of methyl malonate (right). Labels O and O′ on the microconstants in superscript denote the carboxylate groups being protonated, while in subscript (if any) denote the carboxylate group already in protonated form.

Acid–base properties of polyprotic compounds are usually characterized by proton-association (*K*) or -dissociation (*K*_a_) equilibrium constants. These constants are of macroscopic type since they involve the concentration of macrospecies that are defined in terms of the number of bound protons (0 ≤ *i* ≤ *n*), regardless of the site of protonation/deprotonation. For clarity, henceforth protonation constants will be referred to. Also, these *K*_1_, *K*_2_, ..., *K*_*n*_ constants are stepwise (successive) ones because they include one single step of the protonation processes, unlike their cumulative derivatives. Latters are denoted by the β_*i*_ constants that accumulate the product of stepwise constants (β_*i*_ = ∏_*j* = 1_^*i*^*K*_*j*_). The microscopic level acid–base characterization of polyprotic compounds requires species-specific protonation constants or microconstants for short. The macroscopic and microscopic protonation pathways are epitomized with malonic acid in Figure 1. Some examples of macro- and microconstants are
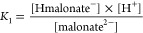
1
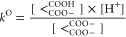
2

Several studies covered the principles of microspeciation (i.e., determination of species-specific protonation constants) based on experimental techniques, such as classical potentiometry or spectroscopy-augmented titrimetric methods.^1−5^ As the number of basic sites grows, the number of microconstants increases exponentially; therefore for macromolecules only site-specific “group” constants are attainable^6^ unless some constraints can be introduced on the microspeciation via symmetry in the compound.^7^ Another approach to minimizing the number of unknown parameters in a microspeciation scheme is the cluster expansion model,^8^ successfully implemented recently for symmetric octaprotic compounds.^9^

A number of papers and book chapters describe the details of the experimental methods used for the determination of macro- and microconstants.^10−14^ There are two fundamental approaches for the determination of microconstants: deductive methods and combined spectroscopic-pH-metric methods. The deductive method relies on the assumption that some protonation constant(s) of a model compound can be equated with certain protonation microconstants of the original compound of interest. These model compounds are relatives of the original compound but contain a reduced number of basic sites. This is usually achieved by replacing a carboxylic acid functional group with the methyl ester or amide derivative, thereby mimicking the protonated carboxylate (i.e., –COOH) moiety. Other common choices are the methyl ether derivatives of phenol or thiol functional groups, making use of the same principle as before. In general, the microspecies to be modeled are minor ones. The modified moiety in the auxiliary compound keeps its protonation state throughout the pH scale and has an effect on the rest of the molecule as similar as possible to that of the parent compound. Note that for an amino basic site no useful model derivative exists, as only the deprotonated mimic of the amine would be beneficial as a model.^15^ The underlying assumption of the deductive method lies in the fact that the electronic (withdrawing or sending) effects of a carboxylic (–COOH) group and a carboxylic methyl ester (–COOCH_3_) group are practically identical as are their Hammett constants.^16−18^ Such close similarity has also been assumed between –COOH and –CONH_2_.^19^ In some cases, the choice between possible derivatives of the main compound to mimic one of the microspecies is not straightforward. To solve this problem, the Hammett approach, as a new deductive tool, was introduced to characterize the minor protonation pathway of the nonsteroidal anti-inflammatory drug tenoxicam.^20^

**Figure 2:**
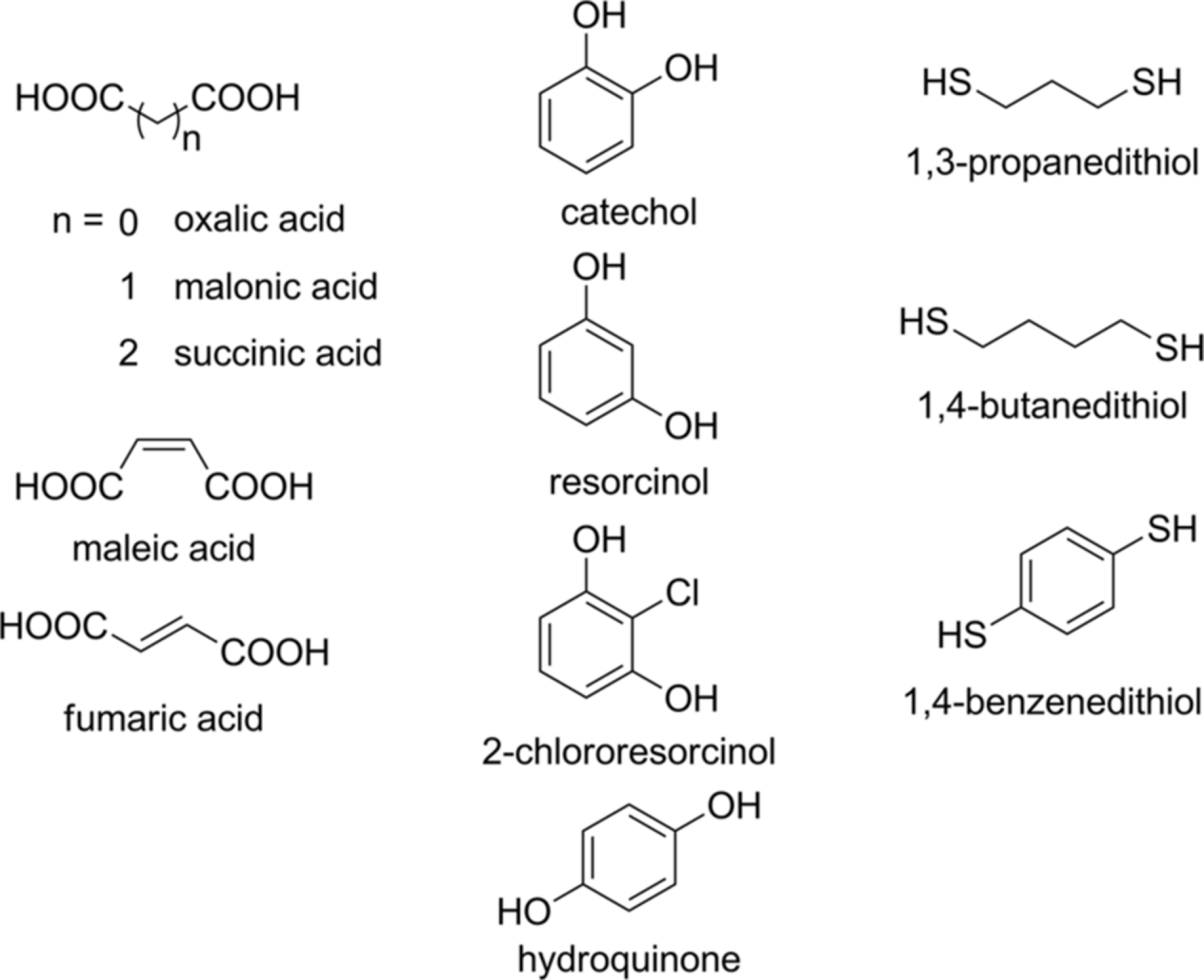
Structural formulas of the parent compounds studied.

However, these small structural differences between the model compound and the investigated compound may result in differences between the intrinsic protonation constant values of the neighboring moieties as well; that is, to some extent the underlying assumption is necessarily violated. This question is still unanswered. In this study, we therefore aimed to gauge the difference between real, unbiased microconstants and microconstants obtained from a deductive method. To quantify the error imposed by the model, the microscopic protonation constant of the original compound has to be determined by a credible method—preferably affording the real, unbiased microconstants, independently from the model compound. An authentic answer is offered by bifunctional, mirror-symmetric compounds, whose unbiased microconstants can be directly obtained from the macroconstants.^7^ For example, the basic, fully anionic form of symmetric dicarboxylic acids (oxalic acid, malonic acid, succinic acid, etc., the structures of the studied compounds are compiled in Figure 2) with two carboxylate sites (designated by O and O′) has the relationships between the *K*_1_ and *K*_2_ macroconstants and the *k*^O^, *k*^O^*’*, *k*_O*’*_^O^, and *k*_O_^O’^ microconstants as follows
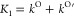
3
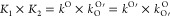
4

Since the carboxylates are identical
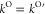
5
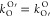
6

It follows that
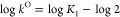
7
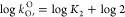
8where log stands for the base 10 logarithm.

The microconstants of symmetric diprotic compounds (depicted in Figure 1 on the example of malonic acid) can be compared to the corresponding protonation constant of a model compound to provide a possible correction factor representing the bias of the model. Such correction factors have been already introduced in the case of histidine, dopamine, and epinephrine.^21−23^
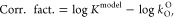
9

One other fundamental parameter determined during the elucidation of microspeciation is the pair-interactivity parameter (ε), which quantifies how much the protonation at one basic site modifies (usually decreases, unless a very seldom cooperativity phenomenon exists in large molecules) the basicity of a neighboring site. The pair-interactivity parameter is a relatively invariant quantity,^24^ i.e., it can be considered multiplicative in polyprotic compounds, as is a useful tool in quantifying the degree of interaction between two basic sites.
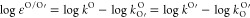
10

By observation of the correction factors described above for the most frequently used model derivatives, the least biased deductive methods can be chosen in the future to determine microconstants. The concentrations of various minor protonation microspecies may influence analytical signals (NMR, UV, etc.) insignificantly; nevertheless, they may be the reactive species in highly specific biochemical reactions, which makes the knowledge of the related microconstants, pair-interactivity parameters important data.

## 2 Experimental Methods

### 2.1 Materials

All chemicals were purchased from Sigma (Merck) and were used without further purification. Deionized water was prepared using a Milli-Q Direct 8 Millipore system.

### 2.2 ^1^H NMR Spectroscopy Measurements

NMR spectra were recorded on a Varian Unity Inova DDR spectrometer (599.9 MHz for ^1^H) with a 5 mm ^1^H{^13^C/^31^P–^15^N} pulse field gradient triple resonance probe head at 298.15 ± 0.1 K. The solvent was H_2_O/D_2_O 95:5 (V/V), and the ionic strength was adjusted to 0.15 mol/L with KCl. The pH values were adjusted with HCl or NaOH and determined in situ by internal indicator molecules (having ca. 1 mmol/L concentration) optimized for ^1^H NMR.^25,26^ The sample volume was 550 μL (containing ca. 5 mmol/L titrand substance), and every sample contained ca. 1 mmol/L DSS (3-(trimethylsilyl)propane-1-sulfonate) as chemical shift reference. The H_2_O ^1^H signal was suppressed with a presaturation sequence; the average acquisition parameters for ^1^H measurements are number of transients = 16, number of points = 65 536, acquisition time = 3.33 s, and relaxation delay = 1.5 s.

### 2.3 pH-Potentiometric Titrations

A 716 DMS Titrino automatic titrator (Metrohm AG, Herisau, Switzerland) with a Metrohm 6.0204.100 combined pH glass electrode was used for the pH-potentiometric titrations under automatic PC control. The electrode was calibrated with an aqueous NBS standard buffer solution. Constant temperature (298.15 ± 0.1 K) was provided by a thermostated double-walled glass cell. Difference titrations were carried out in the absence (blank) and presence of a titrand substance. First, 2 mL of 0.1 mol/L HCl solutions was titrated with 0.1 mol/L KOH. A constant ionic strength of 0.15 mol/L was provided by the presence of KCl. Next, a titrand was added to the same volume of HCl solution and subsequently titrated with KOH. The initial concentration of the titrand substance was around 10 mmol/L in the titrations. Nonlinear parameter fitting provided the protonation constants from the interpolated volume differences.

### 2.4 Statistical Analysis

To analyze the NMR titration data, nonlinear regression was performed using R version 4.0.5^27^ (R Foundation for Statistical Computing, Vienna, Austria) with the function
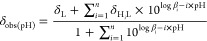
11where δ_L_ is the chemical shift of an NMR nucleus in an unprotonated moiety, δ_H_*i*_L_ is the chemical shift of the same NMR nucleus in the *i*-times protonated species, *n* is the maximum number of protons that can bind to L (the titrand ligand), and log β is the base 10 logarithm of the cumulative protonation macroconstant. The potentiometric titration data were analyzed using the following function
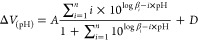
12where *A* is the KOH volume corresponding to one unit of deprotonation and *D* is the experimental correction fitting factor. The standard deviations of log β values from the regression analyses were used to calculate the Gaussian propagation of uncertainty to the other equilibrium constants derived in the Results.

## 3 Results and Discussion

**Figure 3:**
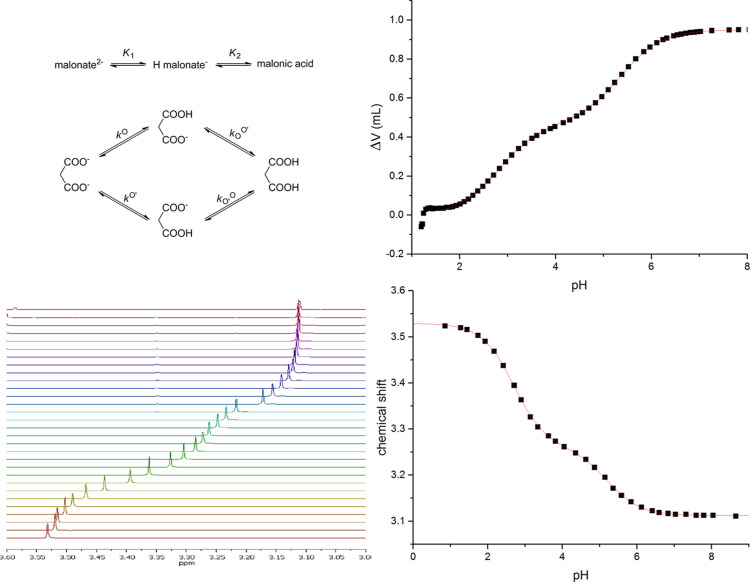
Protonation schemes of malonic acid (top left), potentiometric titration data (top right), recorded ^1^H NMR spectra of malonic acid with pH increasing upward (bottom left), and the fitted chemical shift data (bottom right).

The studied symmetric molecules and their derivatives were titrated using ^1^H NMR-pH titration (except oxalic acid due to the lack of carbon-bound hydrogens) and potentiometric-pH titration (except for the dithiol and catechol derivatives due to oxidation that would bias the results) under identical near physiological conditions (298 K, 0.15 mol/L ionic strength)—sample titration curves are depicted in Figure 3. The determined macroscopic protonation constants (log *K*_1_, log *K*_2_) from the regression analyses together with their standard errors are compiled in Tables 1 and 2. The standard errors of the macroscopic protonation constants from the ^1^H NMR titrations are those of the regression standard errors; in the case of potentiometric titrations, the standard error values are the standard deviation of 3 repeated titration results. Tables 1 and 2 also contain the microscopic protonation constants and interactivity parameters of the parent compounds, calculated using eqs 7 and 8. The protonation constants (log *K*) of the concomitant model compounds are listed next to each parent compound followed by the correction factor, the latter calculated using eq 9. The standard errors of the derived parameters were calculated using the Gaussian propagation of uncertainty.

**Table 1:** Protonation Constants Determined with ^1^H NMR-pH Titrations, Their Related Derived Parameters, and Their Standard Error Values are in Italics^a^

	^1^H NMR-pH titration										
parent compound	macroconstants		microconstants		interactivity par		model compound	protonation const		correction fact	
malonic acid	5.22	0.01	4.92	0.01	1.92	0.01	monomethyl malonate	3.197	0.005	0.196	0.009
	2.700	0.008	3.001	0.008			malonamic acid	3.530	0.005	0.529	0.009
succinic acid	5.18	0.02	4.88	0.02	0.60	0.04	monomethyl succinate	4.38	0.01	0.10	0.03
	3.98	0.03	4.28	0.03			succinamic acid	4.45	0.01	0.17	0.03
maleic acid	5.777	0.004	5.476	0.004	3.38	0.02	monomethyl maleate	2.790	0.002	0.69	0.02
	1.79	0.02	2.10	0.02			maleamic acid	3.556	0.005	1.46	0.02
fumaric acid	4.08	0.03	3.78	0.03	0.55	0.04	monomethyl fumarate	3.178	0.009	–0.05	0.03
	2.93	0.03	3.23	0.03							
catechol	13.26	0.01	12.96	0.01	3.26	0.01	guaiacol	9.913	0.005	0.216	0.007
	9.396	0.005	9.697	0.005							
resorcinol	11.09	0.01	10.79	0.01	1.21	0.01	3-methoxyphenol	9.599	0.009	0.02	0.01
	9.28	0.01	9.58	0.01							
2-chlororesorcinol	10.150	0.007	9.849	0.007	1.60	0.01	2-chloro-3-methoxyphenol	8.15	0.02	–0.10	0.02
	7.950	0.006	8.251	0.006							
hydroquinone	11.83	0.03	11.53	0.03	1.17	0.04	4-methoxyphenol	10.200	0.002	–0.16	0.02
	10.06	0.02	10.36	0.02							
1,3-propanedithiol	10.98	0.03	10.68	0.03	0.67	0.04	S-methyl-1,3-propanedithiol	10.53	0.03	0.52	0.04
	9.71	0.03	10.01	0.03							
1,4-butanedithiol	11.0	0.2	10.7	0.2	0.42	0.22	S-methyl-1,4-butanedithiol	10.41	0.04	0.1	0.1
	10.0	0.1	10.3	0.1							
1,4-benzenedithiol	7.42	0.02	7.12	0.02	1.07	0.03	S-methyl-1,4-benzenedithiol	6.23	0.01	0.18	0.02
	5.75	0.02	6.05	0.02							

a The values for catechol and guaiacol were reported by Mirzahosseini et al. (2018).^22^

**Table 2:** Protonation Constants Determined with Potentiometric-pH Titrations, Their Related Derived Parameters, and Their Standard Error Values are Shown in Italics^a^

	potentiometric titration										
parent compound	macroconstants		microconstants		interactivity par		model compound	protonation const		correction fact	
oxalic acid	4.27	0.01	3.97	0.01	2.39	0.01	monomethyl oxalate	1.64	0.04	0.06	0.04
	1.28	0.01	1.58	0.01			oxamic acid	2.05	0.03	0.47	0.03
malonic acid	5.33	0.02	5.03	0.02	1.91	0.03	monomethyl malonate	3.29	0.01	0.17	0.02
	2.82	0.02	3.12	0.02			malonamic acid	3.58	0.03	0.46	0.04
succinic acid	5.28	0.02	4.98	0.02	0.61	0.02	monomethyl succinate	4.43	0.02	0.06	0.02
	4.07	0.01	4.37	0.01			succinamic acid	4.47	0.01	0.10	0.01
maleic acid	5.87	0.01	5.57	0.01	3.34	0.03	monomethyl maleate	2.72	0.04	0.49	0.05
	1.93	0.03	2.23	0.03			maleamic acid	3.60	0.02	1.37	0.04
fumaric acid	4.18	0.02	3.88	0.02	0.63	0.05	monomethyl fumarate	3.23	0.04	–0.02	0.06
	2.95	0.05	3.25	0.05							
resorcinol	11.11	0.02	10.81	0.02	1.24	0.03	3-methoxyphenol	9.51	0.03	–0.06	0.04
	9.27	0.02	9.57	0.02							
2-chlororesorcinol	10.13	0.02	9.83	0.02	1.58	0.02	2-chloro-3-methoxyphenol	8.13	0.03	–0.12	0.03
	7.95	0.01	8.25	0.01							
hydroquinone	11.52	0.04	11.22	0.04	1.02	0.04	4-methoxyphenol	10.12	0.04	–0.08	0.04
	9.90	0.02	10.20	0.02							

a Values for oxalic acid were reported in the works of Pinching and Bates (1948), and Gelb (1971).^28,29^

**Figure 4:**
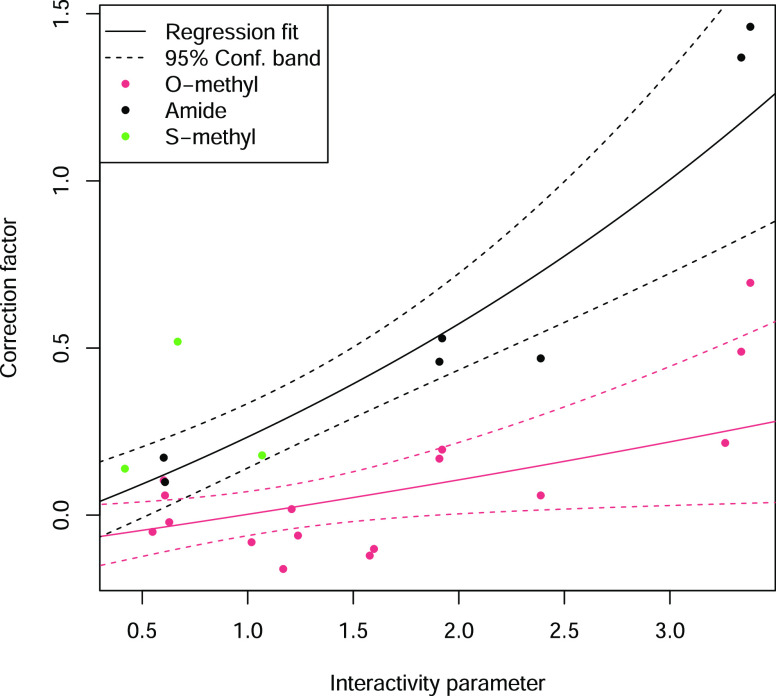
Exponential regression fit of correction factor values as a function of the interactivity parameter of the parent compound. The observed values and fitted curves (grouped by types of model compound) with 95% prediction bands were obtained with a weighted least-squares linear mixed-effects regression model. In the statistical model, the method of measurement (NMR or potentiometry) was not considered and thus data points obtained for the same molecule but from different methods are depicted separately on the graph. Estimates (and standard errors) of *b*_0_ and *b*_1_ are O-methyl −0.09535 (0.05497), 0.09785 (0.03978); amide −0.03225 (0.04870), 0.24239 (0.02999). Note that the data points pertaining to S-methyl model compounds were omitted due to the small group size.

Correction factors show a dependence on the interactivity parameter (log ε) of the symmetric parent compound. To express the relationship, an exponential model has been found appropriate: log(Corr. fact.) = *b*_0_ + *b*_1_ × log ε, where *b*_0_ and *b*_1_ denote the intercept and slope of the linearized model. It is based on the log transform of the correction factor plotted against log ε, and the model was applied with weighted least-squares in which variance is inversely proportional to log ε. The model was fitted on the above data points grouped according to model compound type (“O-methyl” for methyl esters and methyl ethers) to give the result in Figure 4. The diagnostic figures (not shown here) of the above model revealed no anomalies regarding residual homogeneity and normality.

**Figure 5:**
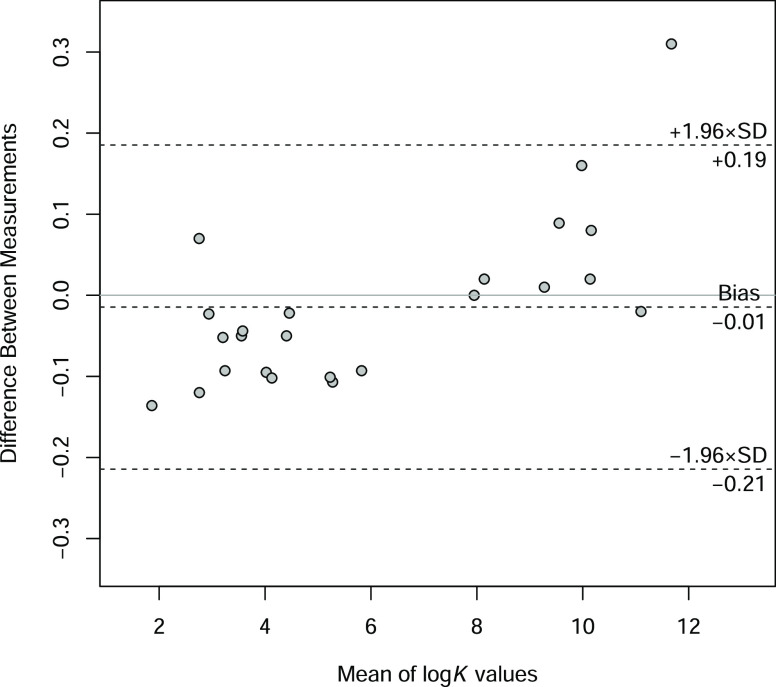
Martin Bland–Altman plot of the protonation constants for the studied compounds obtained with two methods: on the horizontal axis the mean of log *K* values determined by NMR-pH and pH-potentiometric methods is depicted, and on the vertical axis the difference of log *K* values is depicted, i.e., NMR-potentiometry.

To assess the effect of different measurement methods (NMR or potentiometry) on the value of the determined protonation constants, the mean of log *K* values obtained by the two methods for each compound was plotted against the difference in the log *K* values obtained by the two methods. The resulting Martin Bland–Altman plot^30^ is depicted in Figure 5. The effect of the measurement method on the log *K* values was also modeled with a mixed effects model (using the nlme^31^ library) where the parent compound was treated as a random intercept effect. There was no significant effect detectable by the method of measurement: numerator degrees of freedom = 1, denominator degrees of freedom = 23, *F*-value = 0.48825, and *p*-value = 0.4917.

In this work, the species-specific protonation constants obtained from model derivatives of the types O-methyl, ester, amide, and methyl S were evaluated in terms of their precision compared to the true microconstants. For this purpose, symmetric diprotic compounds were chosen for two reasons: (1) This is the sole class of compounds whose microconstants are considered “true” or “unbiased”, since their microconstants can be calculated directly from the macroconstants, without the use of any secondary methods, auxiliary compounds, or assumptions. (2) These compounds are, nevertheless, capable of forming such derivatives that would be the only choices to determine microconstants of nonsymmetric compounds. Comparison of this second type, “derived” microconstants to the “true” ones allows the assessment of the possible bias originating from the derivatization. From the results above, the mean square errors (MSE) and bias^2^ of the correction factor calculated for the model compounds are as follows: O-methyl MSE = 0.058, bias^2^ = 0.008, Var = 0.052; amide MSE = 0.684, bias^2^ = 0.430, Var = 0.297; S-methyl MSE = 0.110, bias^2^ = 0.078, Var = 0.044. The estimates of the S-methyl derivatives are unreliable due to the low number of data points; however, they are mentioned for completeness. There is a strong apparent relationship between the magnitude of the correction factor and the interactivity parameter (log ε) of the diprotic compounds, therefore the above estimates could be misleading. Nevertheless, based on the MSE and bias^2^ estimates, and confirmed by the 95% confidence bands of the fitted models in Figure 4, it is obvious that the O-methyl model is superior to the amide derivatives: the confidence band of the O-methyl model contains Corr. fact. = 0 essentially throughout the entire range of log ε. The results regarding the precision of S-methyl model compounds are inconclusive.

The relationship between the correction factor and the interactivity parameter is best understood as the result of inductive effects (in some cases also mesomeric or steric effects, e.g., in oxalic acid, maleic acid, and catechol) between the basic moieties: the greater the interactivity parameter between two basic moieties, the stronger the distorting effect of a –COOH to –COOCH_3_ substitution on the inherent basicity of the neighboring basic site. It is noteworthy that although the covalent distance in maleic acid is greater between the two basic sites compared to oxalic acid, the interactivity parameter is far stronger in the former compound, due—at least partly—to the double bond with (*Z*) configuration in maleic acid. This phenomenon is also present for catechol^22^ and may be related to the different intramolecular hydrogen bond-forming abilities of the compounds; however, the elucidation of this phenomenon would require further investigation. The fact that O-methyl derivatives of carboxylic acids proved to be a more reliable model compared to the amide counterparts can be best explained by their similar inductive effects and Hammett constants. The OH to NH_2_ substitution perturbs the molecule to a greater extent when regarding atom electronegativities, polarizability, and hydrogen bond-forming abilities. On the other hand, the OH to OCH_3_ substitution occurs one σ bond farther from the neighboring basic site and does not incur as great a perturbation on the electron density of the molecule.

It is noteworthy that when comparing the acid–base parameters obtained by the two methods (Figure 5), although there is no overall bias, there is a clear tendency for protonation constants determined with NMR and in situ pH indicators to underestimate the values compared to potentiometric ones at the <7 log *K* range, and vice versa for the >7 log *K* range. Obviously, there is a difference in the pH determination principle between the two titrimetric methods; furthermore, the combined glass electrode will suffer from bias at pH below 2 and above 12 ranges. It is not yet clear whether the apparent systematic deviance between the two applied methods is due to different measurement principles or if it is mostly variability from measurement noise. A larger number of measurements should be compared to address this question; therefore, for this purpose, we are performing a survey within the literature to compare different methods used for determining protonation constants with a meta-analysis. This result will be published subsequently.

## 4 Conclusions

In conclusion, it can be stated that the O-methyl and S-methyl (as extension from the O-methyl derivatives based on their chemical nature) derivatives are sufficiently good models for the deductive elucidation of microconstants for polyprotic compounds. Nevertheless, in special cases (where an interactivity parameter above 3 is anticipated or an intramolecular H-bond is likely between the basic sites), it is useful to take into account a correction factor to elucidate the set of microconstants. This correction factor can be obtained from the parameters given in the caption of Figure 4, or by finding a simplified symmetric derivative similar to the parent compound.^21^
